# Two Cases of Adult-Onset Esotropia With a History of Medial Rectus Muscle Recession Treated With Botulinum Toxin Type A

**DOI:** 10.7759/cureus.100106

**Published:** 2025-12-26

**Authors:** Hirohito Iimori, Nami Okujima, Atsushi Shiraishi

**Affiliations:** 1 Department of Ophthalmology, Ehime University School of Medicine, Toon, JPN

**Keywords:** acquired comitant esotropia, adult-onset esotropia, botulinum toxin, diplopia, esotropia, medial rectus, ocular alignment, strabismus

## Abstract

Reports on the use of botulinum toxin type A (BTX-A) in patients with esotropia who have previously undergone strabismus surgery are limited. We present two cases of acquired adult-onset esotropia treated with medial rectus recession followed by BTX-A injection and describe the outcomes.

In Case 1, a 49-year-old woman underwent bilateral medial rectus recession for acquired concomitant esotropia. Postoperatively, diplopia resolved, but intermittent recurrence was noted 12 months later. At 26 months postoperatively, BTX-A (2.5 units) was injected into the left medial rectus muscle transconjunctivally under electromyography (EMG) guidance. Diplopia resolved shortly after the injection but recurred three months later, prompting a second injection. Follow-up is ongoing.

In Case 2, a 62-year-old woman with high myopia and mild abduction limitation underwent bilateral medial rectus recession for esotropia, but ocular alignment did not improve. Five months postoperatively, BTX-A (2.5 units) was injected into both medial rectus muscles transconjunctivally under EMG guidance. Although temporary exotropia developed, ocular alignment gradually improved, and diplopia resolved. As of three months after the injection, there has been no recurrence of esotropia, and the patient remains under observation.

In both cases, strabismus surgery and BTX-A injections were performed by the same surgeon. Although the observation period after BTX-A injection was limited to three months in both cases, the treatment was associated with improvement in ocular alignment and resolution of diplopia, while recurrence was noted in one case.

BTX-A injection can be performed using standard techniques, even in patients with a history of strabismus surgery, and may provide temporary improvement in ocular alignment. This report provides illustrative evidence of the feasibility of BTX-A treatment, suggesting that it may be considered as one of the potential options for managing recurrent or undercorrected esotropia following medial rectus recession.

## Introduction

In recent years, the number of patients with acute acquired concomitant esotropia has reportedly increased, particularly among children and young adults [1]. This trend may be associated with the widespread use and prolonged screen time of digital devices such as smartphones and tablets [2-4].

In middle-aged and older adults, acquired esotropia is often attributed to orbital or extraocular muscle imbalances and is associated with conditions such as heavy eye syndrome, sagging eye syndrome, or crowded orbital syndrome [5-7].

Although prism glasses are often used as a conservative treatment for acquired esotropia, they may not sufficiently alleviate symptoms. Furthermore, some patients prefer not to wear glasses. In such cases, botulinum toxin type A (BTX-A) injection into the medial rectus muscle or strabismus surgery may be considered.

BTX-A blocks acetylcholine release at the neuromuscular junction, leading to reversible muscle relaxation. Since the 1980s, it has been used to treat strabismus [8,9]. In Japan, BTX-A was approved in 2015 for the treatment of strabismus in patients aged 12 years or older. Now, BTX-A is employed in various types of strabismus, including acquired esotropia [10,11].

Although BTX-A treatment tends to yield less favorable long-term outcomes than surgery, it is minimally invasive and cost-effective. Some reports suggest that it can be considered as a first-line treatment option in certain cases [12].

Surgical treatment often restores binocular vision and ocular alignment in patients with acquired esotropia [13]. However, esotropia may recur, or surgical outcomes may be suboptimal. Reports on the efficacy of BTX-A in patients with prior medial rectus recession are limited, with most studies focusing on childhood-onset esotropia, such as infantile or accommodative esotropia [14,15]. Its use in adults who have previously undergone medial rectus recession remains underreported, and the clinical outcomes in this subgroup are not well-established. Additionally, the optimal BTX-A dose for esotropia remains under debate [16].

This report presents and aims to describe two cases of adult-onset acquired esotropia treated with medial rectus recession followed by BTX-A injection and discusses their clinical courses and therapeutic outcomes.

## Case presentation

Case 1

A 49-year-old woman had been using hard contact lenses for myopia correction. She began experiencing intermittent diplopia at distance in her 20s, which progressed to constant diplopia in her early 30s. Although prism glasses were prescribed at a local clinic, they did not adequately relieve her symptoms. She was referred to our department for further evaluation and treatment. Her medical history was unremarkable.

**Figure 1:**
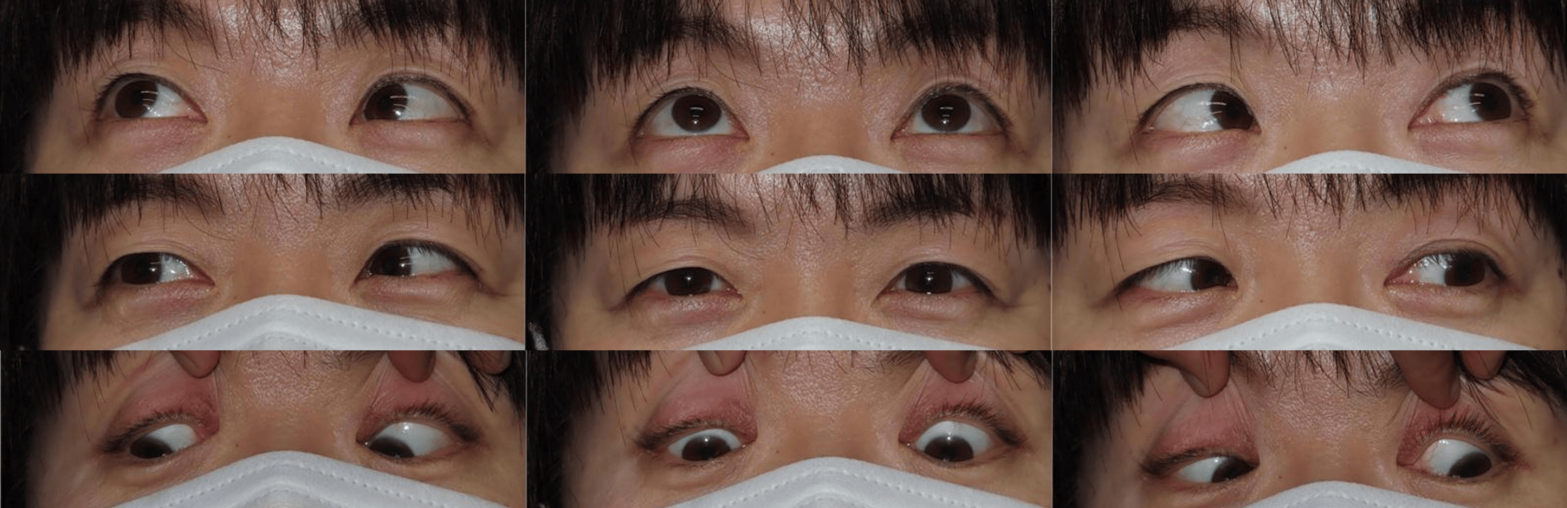
Nine-gaze position photographs of Case 1 No limitation in ocular motility was observed.

At the initial visit, her best-corrected visual acuity was 1.2 decimals in both eyes (right eye: S -3.0 D; left eye: S -3.25 D). Under contact lens correction, alternate prism cover testing revealed 18 prism diopters (PD) of esotropia at distance and 14 PD at near. The Titmus Fly Test showed Fly (+), Animals 3/3, and Circles 9/9. Ocular motility was full, and no intraocular abnormalities were observed (Figure 1). Brain MRI ruled out intracranial pathology, and a diagnosis of acquired concomitant esotropia was made.

Five months later, bilateral medial rectus recession (4.5 mm) was performed under general anesthesia. The recession amount was determined based on the results of a prism adaptation test. Intraoperatively, no muscle contracture or abnormal course was observed, and the surgery was completed without any complications. Postoperatively, diplopia resolved promptly. At 12 months postoperatively, ocular alignment measured 4 PD of esophoria at both distance and near. However, she gradually began to experience diplopia at a distance following prolonged near work.

At 26 months postoperatively, the patient requested BTX-A treatment. A transconjunctival injection of BTX-A (2.5 units) was administered into the left medial rectus muscle by the same surgeon who had performed the strabismus surgery. Under topical anesthesia, a 30-gauge, 25-mm electromyography (EMG) needle was inserted vertically through the nasal conjunctiva toward the dorsal side, avoiding scleral perforation. BTX-A was injected under EMG guidance. During needle insertion, no marked resistance related to conjunctival or muscular adhesions from prior strabismus surgery was encountered, and BTX-A injection could be performed in the same manner as in patients without a history of strabismus surgery.

**Figure 2:**
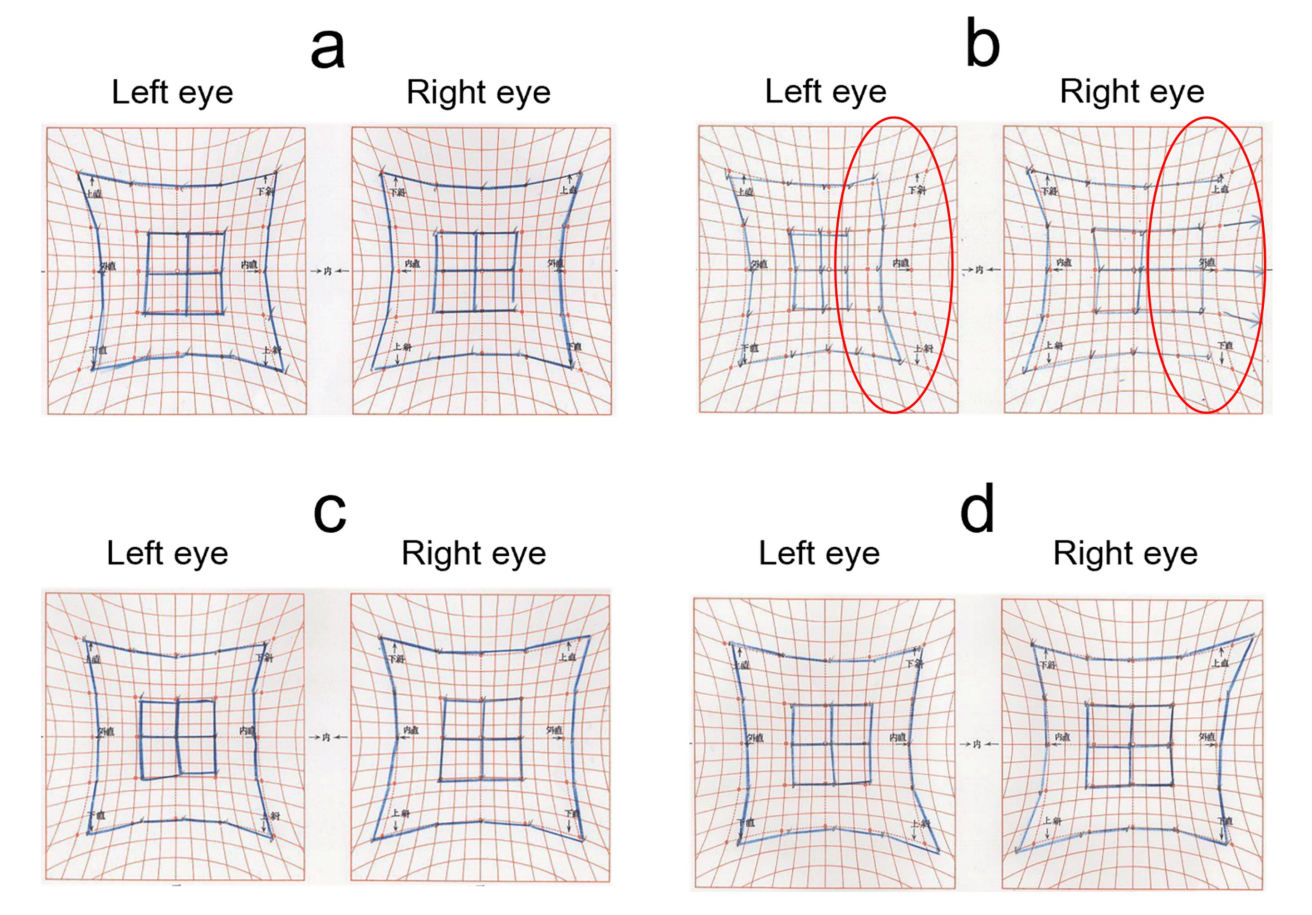
Hess chart progression in Case 1 (a) Before BTX-A injection: Mild esotropic deviation. (b) One week after injection: Adduction limitation in the left eye (red circle). (c) One month after injection: Improvement in adduction and ocular alignment. (d) Three months after injection: Recurrence of mild esotropia. BTX-A = botulinum toxin type A

Pre-injection ocular alignment was 6 PD of esotropia at distance and orthophoria at near. One month later, eye position improved to orthophoria at distance and 6 PD of exotropia at near, with resolution of diplopia. However, 3 months after injection, ocular alignment returned to 6 PD of esotropia at distance and 4 PD of exotropia at near (Figure 2), accompanied by occasional diplopia. A second BTX-A injection (2.5 units) was administered into the left medial rectus muscle, and the patient is currently under observation. The changes in the angle of deviation and the Titmus stereo test results are shown in Table 1.

**Table 1:** Changes in the angle of deviation and the Titmus stereo test results in Case 1 PD = prism diopter; ET = esotropia; XP = exophoria; EP = esophoria; BTX-A = botulinum toxin type A

Time Point	Distance Deviation (PD)	Near Deviation (PD)	Titmus Stereo Test
Pre-surgery	18 ET	14 ET	Fly (+), Animals 3/3, Circles 9/9
1 month after surgery	orthophoria	4 XP	unchanged
12 months after surgery	4 EP	4 EP	unchanged
26 months after surgery (BTX-A injection performed)	6 ET	orthophoria	unchanged
1 month after BTX-A injection	ortho	6 XP	unchanged
3 months after BTX-A injection	6 ET	4 XP	unchanged

The follow-up period for this case was three months after the initial botulinum toxin injection, during which a recurrence of strabismus was observed. However, botulinum toxin injection could be performed in the previously operated muscle using the same technique as in unoperated muscles, and although the reduction in the angle of deviation was temporary, it was achieved, and a second injection could also be administered.

Case 2

A 62-year-old woman with severe myopia (approximately -10.0 D) had been wearing hard contact lenses since the age of 18. She had experienced intermittent diplopia for the previous 10 years, which progressed to persistent diplopia 5 years prior. Although prism glasses were prescribed by a local physician, her symptoms did not improve, prompting her visit to our department. She had undergone bilateral cataract surgery at age 61 and had no other systemic illnesses.

**Figure 3:**
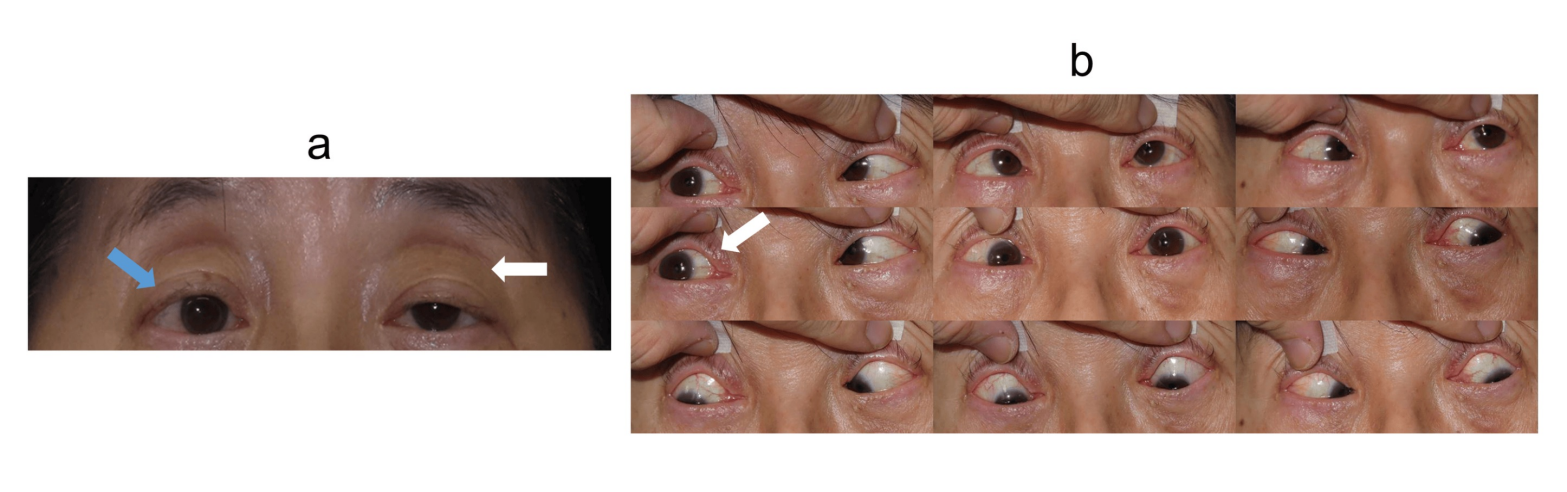
Nine-gaze position photographs of Case 2 (a) Primary gaze: Esotropia in the right eye (blue arrow) and left ptosis (white arrow). (b) Nine-gaze: Mild adduction limitation in the right eye (white arrow).

At presentation, her best-corrected visual acuity was 1.2 decimals in both eyes (right: S -1.75 D, C -1.50 D ×180°; left: S -1.75 D, C -0.75 D ×170°). Alternate prism cover testing revealed 18 PD of esotropia at distance, 8 PD at near, and 3 PD of right hypertropia. The Titmus Fly Test showed Fly (+), Animals 0/3, and Circles 0/9. Mild abduction limitation in the right eye and left ptosis were noted (Figure 3).

**Figure 4:**
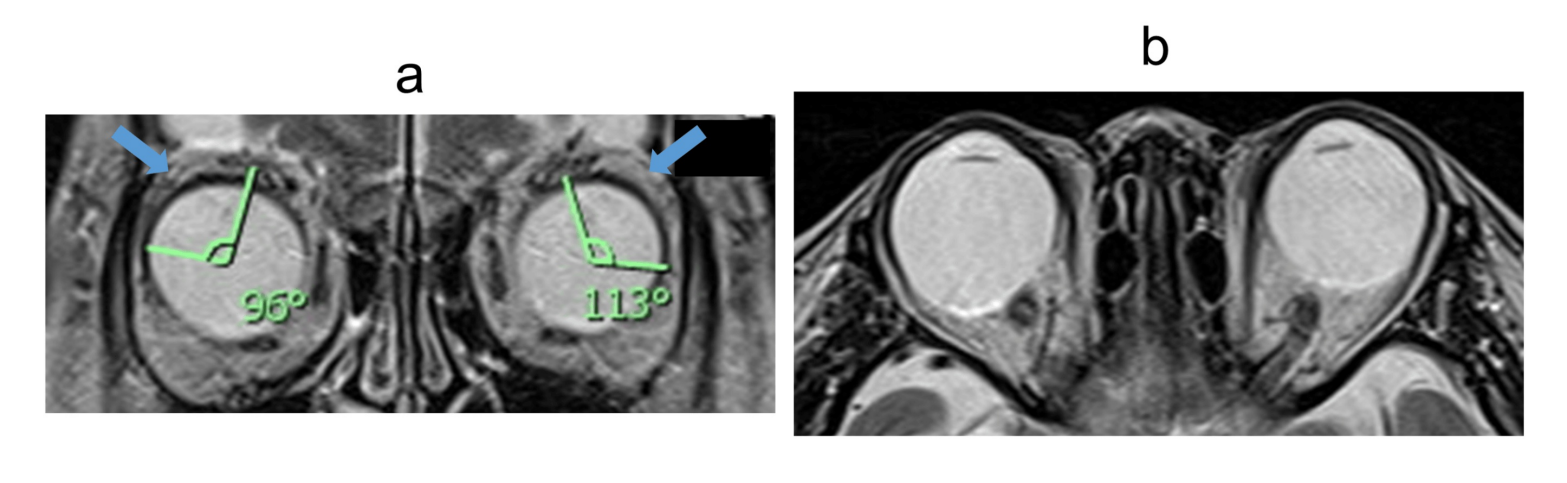
Orbital MRI of Case 2 (a) The angle formed by the centroids of the globe, superior rectus, and lateral rectus was slightly increased (right: 96°, left: 113°). The lateral rectus–superior rectus (LRSR) band was poorly visualized (blue arrow). (b) The globe appeared relatively large in relation to the orbital volume.

Both eyes had intraocular lenses. Fundus examination revealed tessellation and mild retinoschisis in the right eye. Axial lengths were 30.51 mm (right) and 29.60 mm (left). Orbital MRI showed nasal displacement of the superior rectus and inferior displacement of the lateral rectus on coronal sections, along with the absence of the lateral rectus-superior rectus (LRSR) band. The angle between the centroids of the superior rectus, globe, and lateral rectus was mildly increased (right: 96°, left: 113°) (Figure 4). These findings were consistent with esotropia featuring components of heavy eye syndrome, crowded orbital syndrome, and sagging eye syndrome.

Six months after the initial visit, bilateral medial rectus recession (7 mm) was performed. Considering that superior rectus-lateral rectus union surgery might be required in the future if the degree of globe dislocation increases, we chose to perform medial rectus recession as the initial procedure. The surgery was completed in the usual manner, with no intraoperative complications or notable findings. However, ocular alignment did not improve, and diplopia persisted. Five months postoperatively, BTX-A (2.5 units) was injected into both medial rectus muscles by the surgeon who performed Case 1, using the transconjunctival EMG-guided method. In this case as well, needle insertion could be performed in the same manner as in patients without a history of strabismus surgery, and there was no influence of tissue adhesions related to the previous surgery.

Pre-injection ocular alignment measured 20 PD of esotropia at distance, 14 PD at near, and 2 PD of right hypertropia. One week after injection, the patient exhibited 12 PD of exotropia and 2 PD of right hypertropia at distance, and 25 PD of exotropia and 2 PD of right hypertropia at near. Bilateral adduction limitations were observed. Although left ptosis worsened, the patient reported improvement in diplopia, likely due to the ptosis covering the visual axis of the left eye.

**Figure 5:**
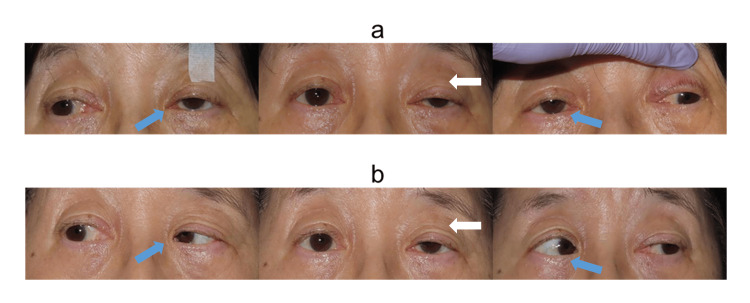
Three-gaze position photographs after BTX-A injection in Case 2 (a) One week after injection: Bilateral adduction limitation (blue arrow) and worsening of left ptosis (white arrow). (b) Three months after injection: Improved adduction (blue arrow) and partial resolution of ptosis (white arrow). BTX-A = botulinum toxin type A

**Figure 6:**
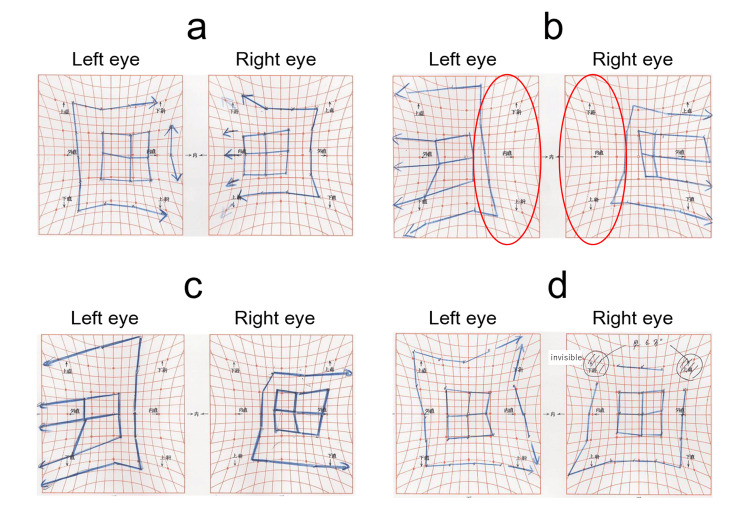
Hess chart progression in Case 2 (a) Before injection: Esotropic deviation. (b) One week after injection: Large exotropic deviation due to bilateral adduction limitation (red circle). (c) One month after injection: Reduced exotropia compared to one week. (d) Three months after injection: Further improvement in ocular alignment.

Over time, both adduction limitation and ptosis gradually improved. At one month post-injection, alignment was 12 PD of exotropia and 3 PD of right hypertropia at distance, and 25 PD of intermittent exotropia at near. At 3 months, alignment improved to 2 PD of esophoria and 3 PD of right hyperphoria at distance, and 8 PD of exophoria and 3 PD of right hyperphoria at near. The Titmus test showed Fly (+), Animals 3/3, and Circles 9/9, indicating resolution of diplopia and recovery of binocular vision (Figures 5-6). The changes in the angle of deviation and the Titmus stereo test results are shown in Table 2.

**Table 2:** Changes in the angle of deviation and the Titmus stereo test results in Case 2 PD = prism diopter; ET = esotropia; XT = exotropia; EP = esophoria; XP = exophoria; RHT = right hypertropia; BTX-A = botulinum toxin type A

Time Point	Distance Deviation (PD)	Near Deviation (PD)	Titmus Stereo Test
Pre-surgery	18 ET, 3 RHT	8 ET	Fly (+), Animals 0/3, Circles 0/9
1 month after surgery	16 ET	12 ET, 2 RHT	unchanged
5 months after surgery (BTX-A injection performed)	20 ET, 2 RHT	14 ET	unchanged
1 week after BTX-A injection	12 XT, 2 RHT	25 XT, 2 RHT	unchanged
1 month after BTX-A injection	12 XT, 3 RHT	25 XT	unchanged
3 months after BTX-A injection	2 EP, 3 RHT	8 XT, 3 RHT	Fly (+), Animals 3/3, Circles 9/9

The follow-up period for this case was three months after the initial BTX-A injection, which is relatively short, and recurrence of esotropia may occur in the future. In such a case, additional BTX-A injections, prism glasses, or strabismus surgery would be potential treatment options.

## Discussion

This case series aimed to evaluate the use of BTX-A in adult patients with recurrent or undercorrected esotropia following prior medial rectus recession, contributing initial evidence suggesting its feasibility and therapeutic benefit in this population. BTX-A induces temporary chemodenervation by inhibiting acetylcholine release at the neuromuscular junction, resulting in reversible muscle weakening, followed by gradual recovery through axonal sprouting and synaptic reorganization [8,9]. The clinical effect of BTX-A in extraocular muscles typically lasts several weeks to a few months, although variability exists depending on dose, muscle condition, and patient-specific factors [16]. Both cases involved adult-onset acquired esotropia with a history of medial rectus recession. Despite prior surgery, BTX-A injection into the previously operated muscle resulted in improvement in ocular alignment. Furthermore, the injection procedure presented no technical difficulties and was comparable to that performed in patients without prior extraocular muscle surgery. Strabismus surgery induces a certain degree of adhesion between the conjunctiva, surrounding tissues, and the rectus muscles, which was expected to make needle insertion during BTX-A injection technically challenging; however, no such difficulty was encountered [17]. These observations are clinically significant.

In Case 1, satisfactory alignment was initially achieved after medial rectus recession; however, diplopia gradually recurred more than two years later. In Case 2, recession alone did not sufficiently correct the esotropia, and alignment remained suboptimal. Although the observation period was short, BTX-A was associated with an improvement in ocular alignment.

Conventional approaches to recurrent or undercorrected esotropia after medial rectus recession include prism glasses, lateral rectus resection, and myopexy of the superior and lateral rectus muscles [6]. However, additional surgery carries potential risks, such as surgical invasiveness, extraocular motility restriction, and overcorrection [15].

In contrast, BTX-A injection can be performed on an outpatient basis, is minimally invasive, and requires simpler postoperative care [10-12]. Moreover, patients with acquired esotropia frequently present with myopia [2-4]. High myopia is associated with an increased risk of future ocular conditions such as glaucoma or retinal detachment [18]. In such patients, it is preferable to minimize conjunctival scarring, as future intraocular surgery may be necessary. From this perspective, BTX-A therapy offers an additional advantage.

There have been few reports on the use of BTX-A in patients with esotropia who have previously undergone medial rectus recession. Tejedor et al. compared outcomes of reoperation versus BTX-A injection in pediatric patients requiring retreatment after esotropia surgery [14]. Among the 24 patients in the reoperation group (20 with esotropia) and 23 in the BTX group (22 with esotropia), the 1-year motor success rate, defined as alignment within 8 PD, was 75% in the surgical group and 69.56% in the BTX group. There was no statistically significant difference between groups, and the authors concluded that BTX-A injection was a rapid and safe treatment option.

Lambert et al. also reported on five pediatric patients with residual esotropia after bilateral medial rectus recession and lateral rectus resection [15]. BTX-A was injected postoperatively, resulting in improvement to <10 PD of esotropia in two cases. Among the three patients with insufficient response, two underwent additional surgery and subsequently developed constant exotropia. The authors noted that BTX-A is unlikely to cause permanent overcorrection.

Notably, all previous reports focused on pediatric-onset esotropia [14,15]. To our knowledge, dedicated adult reports describing BTX-A use after prior medial rectus recession are scarce. According to Bort-Martí et al., surgical treatment demonstrates better outcomes than botulinum toxin injection for strabismus in adults compared with children [19]. Adult extraocular muscles may respond differently to BTX-A than pediatric muscles, as age-related factors, such as increased fibrosis, altered muscle elasticity, and different sensory adaptation, may influence treatment outcomes [20]. Although the underlying reasons are not clearly described in their review, it may be speculated that differences in baseline binocular function and anatomical characteristics could contribute to these age-related distinctions. Variations in the diffusion profile of the agent, potentially influenced by tissue density or structural differences between adults and children, may also play a role.

In the present cases, botulinum toxin was administered to patients with a history of strabismus surgery. Considering that a certain degree of postoperative adhesions and atrophy around the medial rectus muscle is expected, the diffusion of the injected agent and the incidence of complications such as ptosis or vertical deviations may differ from those in patients without prior surgery [17]. Furthermore, in both of the current cases, the medial rectus muscles had been recessed, suggesting that the point reached by the needle tip was located in a portion of the muscle belly closer to the insertion. This change could influence the way in which the drug’s effect manifests. Future studies with carefully standardized inclusion criteria and uniform disease characteristics will be necessary to more precisely evaluate the efficacy of botulinum toxin treatment in patients with a history of strabismus surgery.

The incidence of acquired concomitant esotropia is reportedly increasing, and more patients will likely experience either recurrence after surgery or insufficient correction in the future [1]. Although treatment strategies remain a topic of debate, it is essential to maintain a variety of therapeutic options. Our report demonstrates that BTX-A can be used effectively, even in previously operated muscles, and the injection can be performed using the same technique as before surgery. Despite its temporary nature, BTX-A provided significant improvement in alignment with minimal invasiveness.

This report is limited by the small number of cases, the absence of a control group, and the short-term follow-up, which preclude any conclusions regarding the efficacy or generalization of outcomes. The findings should therefore be interpreted as illustrative rather than confirmatory evidence. Further prospective studies with larger adult cohorts and standardized dosing protocols are warranted to clarify whether prior medial rectus surgery influences the indication, timing, dosage, or duration of BTX-A effects in the treatment of esotropia. In addition, future research should investigate how repeated BTX-A injections may affect treatment response and long-term stability.

## Conclusions

BTX-A injection is one of the minimally invasive treatment options for strabismus, including esotropia, which can be performed on an outpatient basis. Our findings suggest that, even in patients with a history of medial rectus recession, BTX-A can be administered using the same technique as in patients without prior surgery and may provide short-term improvement in ocular alignment. This report offers illustrative evidence of the feasibility of BTX-A treatment as a potential therapeutic option for recurrent or undercorrected esotropia after medial rectus recession. Further accumulation of cases is needed to better define its indications and the stability and duration of its therapeutic effect.
